# Analysis on the Influence of Component Ratio on Properties of Silica/Montmorillonite Nanocomposites

**DOI:** 10.3390/ma11112074

**Published:** 2018-10-24

**Authors:** Jun Jiang, Jinzhen Cao, Wang Wang, Changtong Mei

**Affiliations:** 1 College of Materials Science and Engineering, Nanjing Forestry University, Nanjing 210037, China; jiangjun116@foxmail.com; 2 MOE Key Laboratory of Wooden Material Science and Application, Beijing Forestry University, Beijing 100083, China; wangwang_1987@bjfu.edu.cn

**Keywords:** montmorillonite, silica, nanocomposites, organic/inorganic hybrid, component ratio, characteristic analysis

## Abstract

Silica/montmorillonite (MMT) nanocomposites (SMCs) were prepared by generating SiO_2_ nanoparticles on an MMT surface using an organic/inorganic hybrid technique with different ratios of tetraethylorthosilicate (TEOS) to MMT (10:1, 20:1 and 40:1). The hydrolysis and polycondensation reactions were controlled by TEOS when it was incorporated into the internal space of the MMT. The delamination and intercalation of the MMT layers were closely related to the TEOS/MMT ratio. The surface chemistry and particulate morphology, thermal properties, pore structure and hygroscopicity of nanocomposites were investigated. The results showed that silica nanoparticles could be intercalated into a layered MMT and induced a high specific surface area (~474 m^2^·g^−1^). At a lower ratio (10:1 and 20:1), the dispersed layers could be created from the stack MMT layers and incorporated into a silica matrix, resulting in an increased thermal stability and a decreased pore size. A higher ratio (40:1) caused the intensive self-condensation of the silanol groups, leading to a negative effect on the sol penetration to the MMT. The hydrophilicity of the SMCs increased significantly due to the synergistic effect of the hydroxyl groups and pore structure caused by silica incorporation. A mechanism concerning the effect of component ratio was also proposed for synthesizing this nanocomposite based on the research results. The potential applications of this heterostructured nanocomposites could be summarized as a desiccant, functional fillers, and pollutant disposal.

## 1. Introduction

Composites prepared by organic and inorganic components have prospectively synergistic properties. Clays, as one of the inorganic materials, have been extensively used in preparing hybrid composites. They can provide advantageous physicomechanical properties to the composites by using as a reinforcing agent or by reducing the number of polymers used as fillers [1,2]. Layered materials, such as layered silicates, are potentially well adapted for tailoring polymer nanocomposites due to their high in-plane strength, high aspect ratio and stiffness [3,4]. Montmorillonite (MMT) is a widely used layered clay for synthesizing polymer composites because it occurs ubiquitously in nature and can be obtained in mineralogically pure form at low cost. These composites, even at a low clay loading, are equipped with improved gas barrier properties, thermal stability and mechanical properties [5,6]. However, the efficiency of the clay (e.g., MMT) to improve the polymer properties is primarily determined by its dispersion in the polymer matrix. The van der Waals attraction makes the MMT particles easier to aggregate and it is difficult to produce stable and uniform dispersions in the polymer [7]. Therefore, the interfacial incompatibility between inorganic clay and organic polymers determines the final properties of the composites.

On the other hand, clays can adsorb heavy metals via ion exchange reactions and by the formation of inner-sphere complexes through ≡Si-O- and ≡Al-O- groups at the edges of clay particles [8]. However, the limitation of this adsorption is the lower loading capacity and relatively smaller metal ion binding constants [9]. To overcome this drawback, the covalent grafting of clays with reagents of metal-chelating has been explored to increase the metal binding capacities of clays. However, a low silanol density on the clay surface limits the performance of the covalent grafting (e.g., thiol functionalities grafting) [10]. Compared with the grafting reaction on the clay surface, the covalent grafting on silica showed better loading capacities and affinities for selected metal ions, which was attributed to the higher density and accessible hydroxyl groups on the silica surface [11].

Hence, the surface properties of clays (e.g., MMT) have a significant effect on its applications. The most common approach applied to modified clay surfaces is to increase the interlayer spacing and to produce a relatively favorable organophilic property using a cation exchange reaction with alkylammonium [12,13]. However, it still cannot change the density of the silanol groups on the clay surface for further modification. Moreover, when it is used as pollution sorbents, the reversible and unselective adsorption of metals via ion exchange or physisorption still limits their environmental applications, which should show strong affinity toward the target metals and irreversible binding to remove the metal from the substances [14]. Sol-gel modification seems to be an alternative to improve the surface reactivity of clay minerals and therefore overcome these limitations. Organosilane reagents, used as a silica source, can attach onto a clay surface through a condensation reaction during the grafting process. Owing to the incorporation of silica, the silanol groups can be grafted on the clay surface simultaneously, which provides more reactive sites for further grafting or functionalization. Besides, the silica particles were also useful fillers used in the manufacturing of composites to improve their mechanical properties, durability and thermal stability [15,16]. Up until now, several researchers have focused on this kind of inorganic-inorganic nanocomposites, most of which attempted to investigate the effect of different alkoxysilanes or organoclays on textural properties and formation of gelation behaviors [17,18]. However, for pristine MMT, there is little discussion available in the literature about the influence of the ratio of alkoxysilane to MMT on the hydrolysis and polycondensation reactions, which could finally give rise to varied heterostructure and affect their properties. For instance, delaminated materials derived from these compounds can be used as a catalyst, reinforcing agents and electroactive materials [19,20,21]. Therefore, it is essential to examine the ratio of precursor to MMT and to find the proper proportion for the preparation of nanomaterials in order to achieve the appropriate properties for practical applications.

Herein, for the further application of MMT either as reinforcing fillers or as adsorbents, heterostructured materials involving MMT and silica nanoparticles were synthesized. This study focused on the influence of the component ratio on the properties of silica/MMT nanocomposites. Tetraethylorthosilicate (TEOS) was applied as the source of silica, the sol-gel method was used to prepare the silica/MMT nanocomposites. This procedure can be accomplished by the reaction between the hydrolyzed TEOS and -OH groups on the MMT surface. In addition, the redundant Si-OH groups on the surface of the composite from the silica can be accessible for further chemical reactions. The hygroscopicity was characterized using the dynamic vapor sorption (DVS) method, which can provide an approach to evaluate the effectiveness for the incorporation of hydroxyl groups and to reveal the potential application of this nanocomposite in desiccant fields. The overall performances were characterized by Fourier transform infrared spectra (FTIR), X-ray diffraction (XRD), thermogravimetric analysis (TGA), differential scanning calorimetric analysis (DSC), scanning electron microscope (SEM), transmission electron microscopy (TEM), and the nitrogen adsorption test.

## 2. Materials and Methods

### 2.1. Materials

Na-MMT (PGV; Naocor Inc., Chapel Hill, NC, USA) was provided by East West Company, Beijing, China. The mean interlayer distance of Na-MMT was 1.42 nm and the cation exchange capacity was 145 mmol per 100 g. Tetraethylorthosilicate (TEOS), absolute ethanol and acetic acid were provided by Beijing Chemical Reagent Co. Ltd. (Beijing, China).

### 2.2. Preparation of Silica/MMT Nanocomposites

Heterostructured nanocomposites were prepared by a heterocoagulation technology involving the sol-gel method. Three mass ratios of TEOS to MMT (10:1, 20:1 and 40:1) were used to assess the influence of the composition on the properties of the silica/MMT nanocomposites, which were labeled as SMC-10, SMC-20 and SMC-40, respectively. TEOS and ethanol were mixed at a constant molar ratio of TEOS to ethanol (1:1). A certain amount of MMT clay calculated according to the determined component ratio was dispersed in deionized water under magnetic stirring at room temperature. Thereafter, the above two solutions were mixed to get a homogenous dispersion. The pH value of the mixture was adjusted to 2–4 with acetic acid. Then, the mixture was stirred at a speed of 3000 rpm for 4 h at 40 °C. The mixture was sealed to avoid the evaporation of ethanol. Silica gel without MMT was also prepared by the hydrolysis and condensation of TEOS in the molar ratio 1:1:4 (TEOS:ethanol:water). The resultant products were dried at 80 °C to generate the silica/MMT nanocomposites.

### 2.3. FTIR Analysis

The change in surface chemistry, including the functional groups of the samples, was investigated using an FTIR spectrometer (Vertex 70v, BRUKER, Billerica, MA, USA). The samples were prepared by the KBr pellet method (Sample/KBr = 0.002).

### 2.4. X-ray Diffraction Analysis

XRD analyses were performed on an X-ray diffractometer (X-ray 6000, Shimadzu, Kyoto, Japan). The X-ray beam was Cu-K*α* (λ = 0.154nm) radiation, operated at 40 kV and 30 mA. The diffractograms were scanned in the 2*θ* range of 2–10°. All the samples were oven-dried before the analysis.

### 2.5. Thermal Characterization

The thermal stability was evaluated by TGA (TG300, SEIKO Instruments, Chiba, Japan). The temperature changed from 30 to 900 °C at 10 °C /min in an N_2_ atmosphere. Prior to the test, all the samples were dried to a consistent weight at 103 ± 5 °C.

DSC (Q2000, TA Instruments, New Castle, DE, USA) was conducted by heating the samples from −50 to 300 °C at 10 °C/min under a constant N_2_ atmosphere.

### 2.6. Particulate Morphology

The surface morphologies of the nanocomposites were observed by SEM (S-3400, Hitachi, Tokyo, Japan). All the samples were dried at (103 ± 5 °C) before testing. Then, prior to observation, a thin layer of Pt-Pd was sputtered onto the samples. For analyzing the dispersion of the nanocomposites, a TEM test was conducted in a JEM 1200 EX П microscope (JEOL, Tokyo, Japan). Prior to the TEM analyses, the sample (2 mg) was dispersed in deionized water with sonication treatment (100 W; 20 min). Thereafter, a drop of the dispersion was applied to a copper grid with a thin carbon film. The remaining particles on the film, after water evaporation, were tested by TEM.

### 2.7. Pore Characteristics

The porous characteristics of MMT and silica/MMT nanocomposites were tested by N_2_ adsorption experiments on an Autosorb-iQ, automatic analyzer (Quantachrome, Boynton Beach, FL, USA) at 25 °C. The specific surface area and pore size were calculated using the Bruanuer-Emmett-Teller (BET) method based on N_2_ adsorption isotherm data [22]. All the samples were degassed at 100 °C for 6 h before the test.

### 2.8. Moisture Adsorption

The moisture adsorption behaviors were determined using DVS (IGAsorp, Hiden Isochema Ltd., Cheshire, UK). The test method with specific details is referred to in our previous study [23]. 

## 3. Results and Discussion

### 3.1. Characteristics of Surface Chemistry

**Figure 1:**
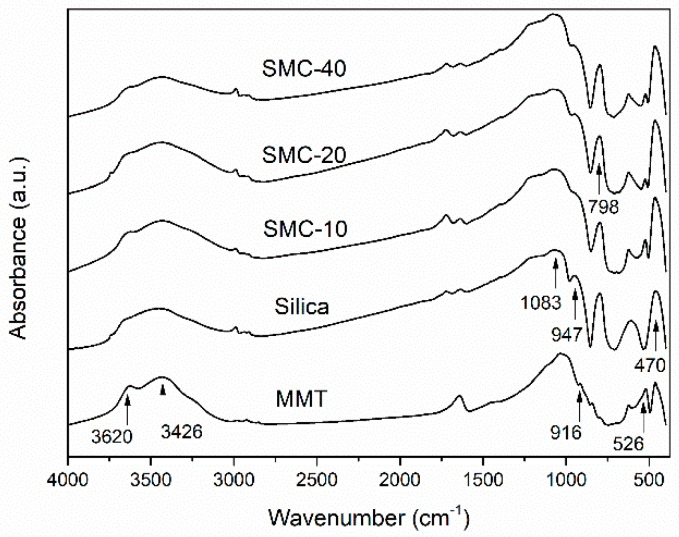
Fourier transform infrared spectra (FTIR) spectra of montmorillonite (MMT), silica and silica/MMT nanocomposites.

The FTIR spectra of MMT, silica and silica/MMT nanocomposites are shown in Figure 1. For MMT, the band at about 3620 cm^−1^ corresponded to the inner hydroxyl groups, lying between the tetrahedral and octahedral sheets [24]. While the bands at 3426 cm^−1^, 916 cm^−1^ and 526 cm^−1^ were associated with the main -OH stretching, Al-Al-OH deformation and Al-Mg-OH deformation, respectively [24,25]. The absorbance at 1083 cm^−1^, 798 cm^−1^ and 470 cm^−1^ was assigned to Si-O and Si-O-Si, respectively [26]. The absorbance at 947 cm^−1^ was associated with the bending vibration of silanol groups (Si-OH) on the silica surface [27]. Some obvious changes could be found in the spectra of silica/MMT nanocomposites. The intensity of the peaks for SMC at 3620 cm^−1^, 916 cm^−1^, 526 cm^−1^ and 947 cm^−1^ decreased when compared to the MMT and silica, indicating that the -OH groups on the MMT surface could react with the -OH groups on the silica surface. This finding was consistent with the discussion in a previous study that demonstrated the covalent bonds between organosilanes and smectite-type clays [28]. Furthermore, the broadening of the band between 3750 cm^−1^ and 3055 cm^−1^ suggested that hydrogen bonds could exist. This was ascribed to the influence of hydrogen bonds on -OH vibrational energy [29]. On the other hand, the peaks at 1083 cm^−1^, 798 cm^−1^ and 470 cm^−1^ appeared in the composites, suggesting that the silica was successfully incorporated with the MMT clay. Compared to SMC-20, the peak intensity at 526 cm^−1^ for SMC-40 showed no further changes, suggesting the self-condensation of hydrolyzed TEOS existed when excessive TEOS was applied. This is consistent with the result reported in our previous study of silane modified silica particles [23]. Similarly, it has been reported that the condensation reaction between the silanol groups could be faster than chemical grafting when more silane was added for surface modification [23,30].

### 3.2. XRD Analysis

**Figure 2:**
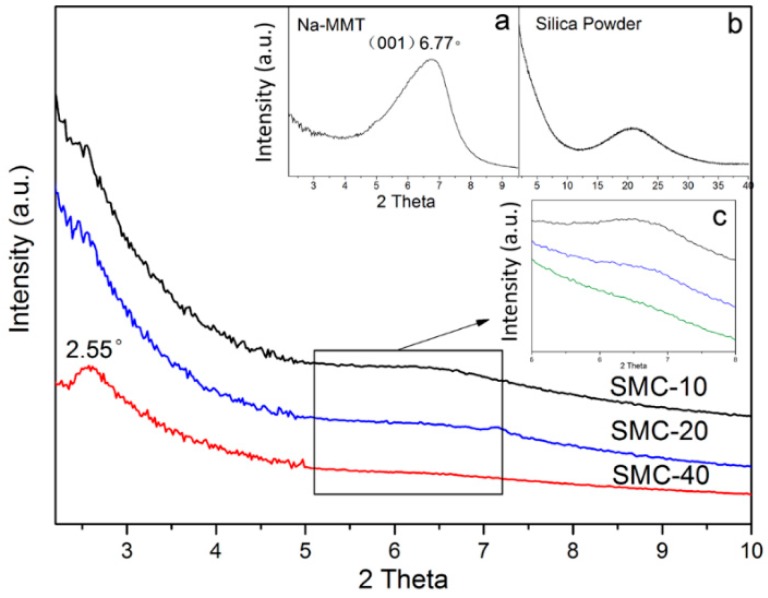
X-ray diffraction (XRD) patterns of MMT, silica and silica/MMT nanocomposites.

The XRD patterns of MMT, silica and silica/MMT nanocomposites are shown in Figure 2. When considering Bragg’s law, the interlayer distance of MMT can be calculated from the (001) lattice plane diffraction peak at 2*θ* = 2–10°.

$$\mathrm{Bragg}’s\;\mathrm{law}:\;d=\frac{n\lambda }{2\sin\theta }$$

where d is interlayer distance value between the (001) plane, n indicates the integer wavelength number (*n* = 1), λ represents the X-ray wavelength, and *θ* is the maximum diffraction angle. After modification, the peaks at 2*θ* = 6.77° (Figure 2a) decreased correspondingly and a new peak at 2*θ* = 2.55° for the silica/MMT composites was observed especially for SMC-40. The former one shows that the interlayer distance of pristine MMT was 1.31 nm and the later one was 3.45 nm due to the intercalation in the galleries of MMT. There was no diffraction peak at 2*θ* = 2–10° for silica but it appeared at ~22° (Figure 2b), indicating a typically amorphous silica structure (Si-O short order structure) [31]. Moreover, the intensity of the (001) diffraction peaks for the silica/MMT composites decreased steadily with the increasing TEOS/MMT ratio (Figure 2a,c), suggesting that the stacking of the silicate sheets became disordered in the c-direction. This was due to the delamination of the layered MMT [20]. The clays can be easily exfoliated in hydrophilic polymers, which is similar to silica sol to some extent. However, SMC-40 shows a different structure compared with other composites, the (001) reflection appeared at lower diffraction angles than that in the pristine MMT. This indicated that the silica sol preferred intercalation rather than delamination and the interlayer distance increased from 1.31 nm to 3.45 nm. In this case, only a part of the silica sol could accede to the interlayer space of MMT and it was not enough to create a complete delamination of the MMT. A higher ratio formula (40:1) caused more self-condensation of Si-OH, resulting in more nucleation centers before accessing the interlayers. This was negative to the penetration of silica and the delamination of MMT. Therefore, the TEOS/MMT ratio could govern the structure of the nanocomposites. In addition, the intercalation did not progress when a higher ratio was applied due to the generation of more nucleation centers at the exterior of MMT particles. These observations were consistent with the FTIR results.

### 3.3. Thermal Properties

**Figure 3:**
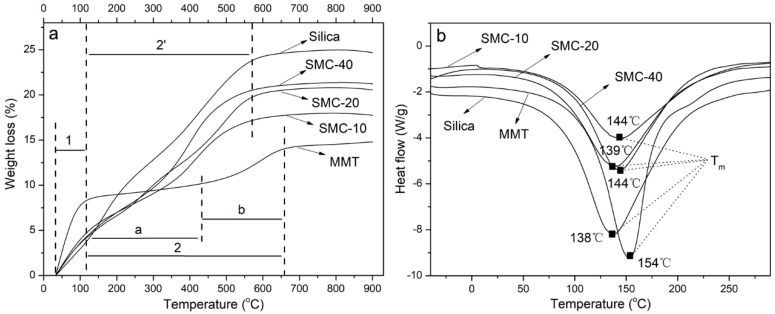
Thermogravimetric analysis (TGA) curves (**a**) and differential scanning calorimetric analysis (DSC) curves (**b**) of MMT, silica and silica/MMT nanocomposites.

The thermal stabilities of the silica/MMT nanocomposite were determined by thermogravimetric analysis. According to the weight loss change, the TGA curves were mainly divided into two stages (stage 1 and 2). As shown in Figure 3a, the initial weight loss of the MMT (stage 1) was observed between 30 and 110 °C. This could be ascribed to the loss of adsorbed water. In the second stage (stage 2), after the mitigatory process (2a), a clear decomposition started at around 450 °C and ended at about 650 °C (b), which was caused by the further loss of water and the dehydroxylation of the MMT. For silica and silica/MMT composites, the initial weight loss, from 30 to 110 °C (stage 1), corresponded to the evaporation of residual free water and ethanol [32]. The second weight loss stage, from ~110 to 550 °C (stage 2’), was due to the loss of bound water present in the voids between the particles as well as the dehydration condensation of Si-OH in the silica or silica/MMT composites. Compared to MMT, silica showed sensitivity to thermal decomposition owing to the existence of more -OH groups on the surface. Therefore, as the ratio of TEOS increased, the stability of silica/MMT showed a decreasing trend. This trend was relatively significant from SMC-10 to SMC-20. However, it also indicated the positive effect of MMT on the thermal stability.

The DSC curves for MMT, silica and silica/MMT composites are depicted in Figure 3b. The MMT clay and heterostructure nanocomposites show different melting endothermic peaks (T_m_). The pristine MMT clay showed the highest melting endotherm, at 154 °C, while the lowest was found at 138 °C for silica. With the incorporation of silica, the intensity of the endothermic melting peaks of the silica/MMT nanocomposites decreased and a change in the melting endotherm was observed, indicating that the combination of MMT and silica developed a new melting characteristic. Similar results were found in the study of clay aerogel/poly (vinyl alcohol) composites [33]. The results of the thermal calculations showed that the △H value increased with a decrease in the MMT loading. The DSC curves show a significant change in the △H values (silica: 325 J/g, MMT: 193.7 J/g, SMC-10: 195.6 J/g, SMC-20: 213.5 J/g and SMC-40: 230 J/g). For silica/MMT nanocomposites, these increased from a lower value for SMC-10 to a higher one for SMC-40. 

### 3.4. Characteristics of Particulate Morphology

**Figure 4:**
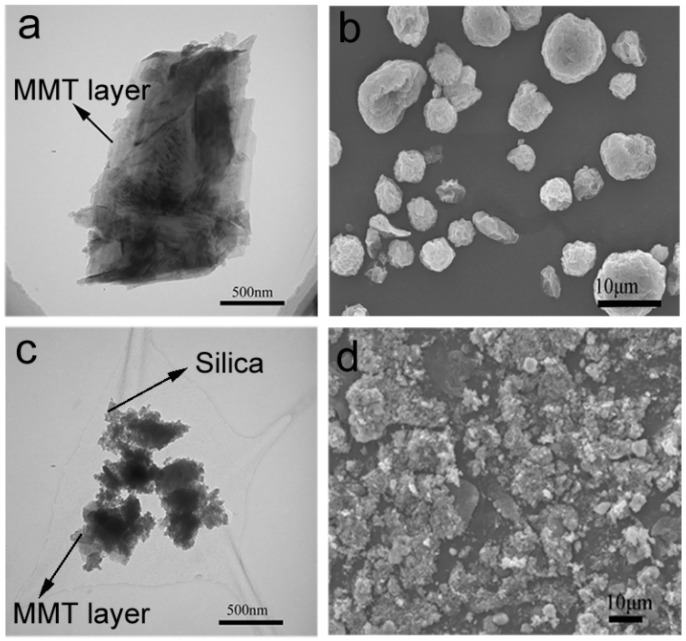
Transmission electron microscopy (TEM) and scanning electron microscope (SEM) images of MMT and silica/MMT nanocomposites. (**a**) TEM image of MMT; (**b**) SEM image of MMT; (**c**) TEM image of SMC-20 and (**d**) SEM image of SMC-20.

Notably, the addition of MMT could reduce the gel time compared with TEOS itself in the aqueous solution. It was considered that the gel could form quickly when the condensation occurred between the Si-OH groups from the hydrolyzed TEOS and -OH groups located on the MMT surfaces. The morphology of the MMT and silica/MMT nanocomposites was characterized by TEM and SEM, respectively (Figure 4a–d). The images confirm the disaggregation of layered MMT and the creation of loosened silica/MMT nanocomposites with heterostructures. Those noncompact heterostructures could contribute to the porosity of the resulting nanocomposites. The silica nanoparticles were assembled in the MMT layers, giving rise to disordered heterostructures. For pristine MMT, the layered structure and sphere agglomeration were observed. As shown in Figure 4c, the composite shows sponge-like materials (agglomerates) due to the silica network incorporated into the swollen MMT [34]. Silica particles with a nanoscale have a high thermodynamic surface energy. It is easy for them to aggregate spontaneously to reach a stable state [35]. A similar structure was reported owing to the condensation reaction between organosilanes and polyhydroxy particles [23]. Thus, the disorder in the stacking of the dispersed clay layers was probably ascribed to the incorporation of the silica particles around them. Moreover, the swelling of the pristine clay in the aqueous layer helped the incorporation of the silica sol and the occurrence of subsequent condensation in the interlayers. In addition, the acidic condition of hydrolysis and condensation reaction favored the formation of smaller colloidal particles, promoting the infusion of silica sol to the galleries of MMT [36].

### 3.5. Characteristics of Pore Structure

**Figure 5:**
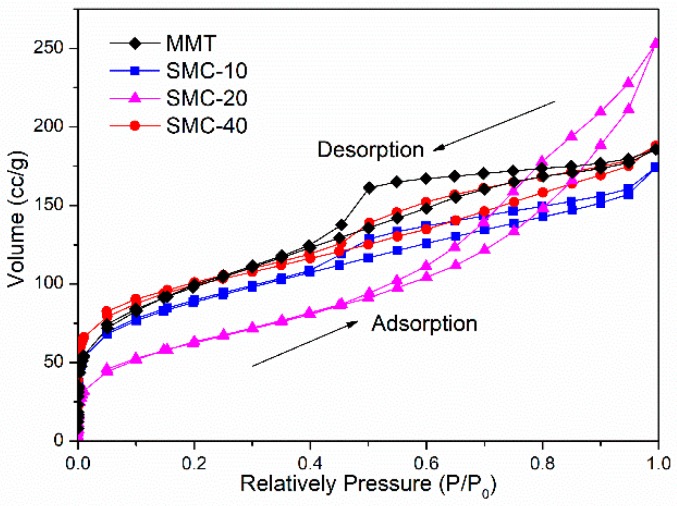
N_2_ adsorption/desorption isotherms of MMT and silica/MMT nanocomposites.

The N_2_ adsorption/desorption isotherms of the pristine MMT and silica/MMT nanocomposites are shown in Figure 5. According to IUPAC classification, all of the isotherms were in the shape of the type II classification [37]. However, differences in the shape of hysteresis loops were evident. The MMT shows a typical Type-H4 hysteresis loop, owing to the narrow slit pore resulted from the layered structure of MMT. It has been described as a reversible type II isotherm for many clay minerals resulting from macroporous aggregates [38]. While the isotherm of SMC-20 shows a Type-H3 hysteresis loop due to the loose agglomerates of nanoparticles [39], indicating the aggregation of silica particles dominated the microstructure characteristics of this nanocomposite. For SMC-10 and SMC-40, the hysteresis loops resemble that of the MMT but with different degrees of hysteresis loops. This is because of the different extents of intercalation or delamination for MMT. Compared to SMC-10, the hysteresis loop of SMC-40 increased again and nearly show the same shape as compared to the MMT, suggesting the increase in TEOS content is against the tendency of the delamination process. This is because the fast hydrolysis of TEOS resulted in more Si-OH groups or silica network on the exterior of the interlayer region of clay. However, the galleries of MMT remain inaccessible and the obtained BET surface area in SMC-40 showed a smaller value compared with that for SMC-20.

Table 1 lists the physical parameters of MMT and silica/MMT nanocomposites. The structure changed obviously due to the generation of an intercalated silica network, exhibiting a notable increase in specific surface area and a decrease in pore size compared to the pristine MMT clay. The BET surface area increased from 73 m^2^·g^−1^ to a maximum of 474 m^2^·g^−1^ after silica incorporation, resulting from the attachment of silica on the internal surface of MMT to form loose agglomerates with a micropore and mesopore network structure. This dramatic increase could be explained from two aspects: (1) the sol-gel process induces a delamination or at least an intercalation of the MMT layers, which makes the internal surface of the MMT layers accessible and (2) the presence of amorphous silica nanoparticles results in a higher specific surface area [23]. Both of these reasons contributed to the decreasing pore size, suggesting the relatively uniform dispersion of delaminated MMT in the silica matrix. In terms of these observations, the silica/MMT nanocomposites at suitable molar ratios have the potential to be used as a desiccant or pollutant adsorbent.

### 3.6. Hygroscopicity

**Figure 6:**
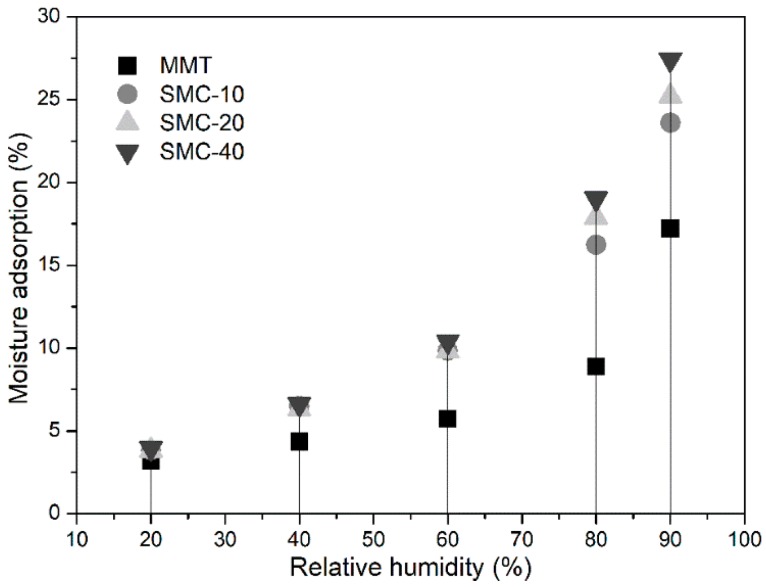
Moisture adsorption of MMT and silica/MMT nanocomposites.

The hygroscopicity of MMT and silica/MMT nanocomposites at 25 °C was investigated. The change of moisture adsorption increased with the increasing relative humidity (RH) (Figure 6). This trend can be classified as Type II [37,39], indicating that the multilayer adsorption occurred by a strong interaction between the adsorbent and adsorbate. The moisture adsorption of SMC was higher than that of MMT, suggesting more sorption sites (-OH groups) were available in SMC compared with that in MMT. In addition, this was attributed to the decreasing pore size, which provided more specific surface area. Both of them showed a synergistic effect on the equilibrium values of moisture adsorption [40]. Obviously, the moisture adsorption increased rapidly within the RH range from 60 to 90%, which was related to the capillary-condensed moisture at higher RH levels [41]. The hygroscopicity of the nanocomposites increased with the increasing ratio of TEOS to MMT, suggesting an improved hydrophilicity of SMC due to the increase in the number of sorption sites (-OH) for moisture adsorption provided by silica particles.

### 3.7. Synthesis Mechanism

**Figure 7:**
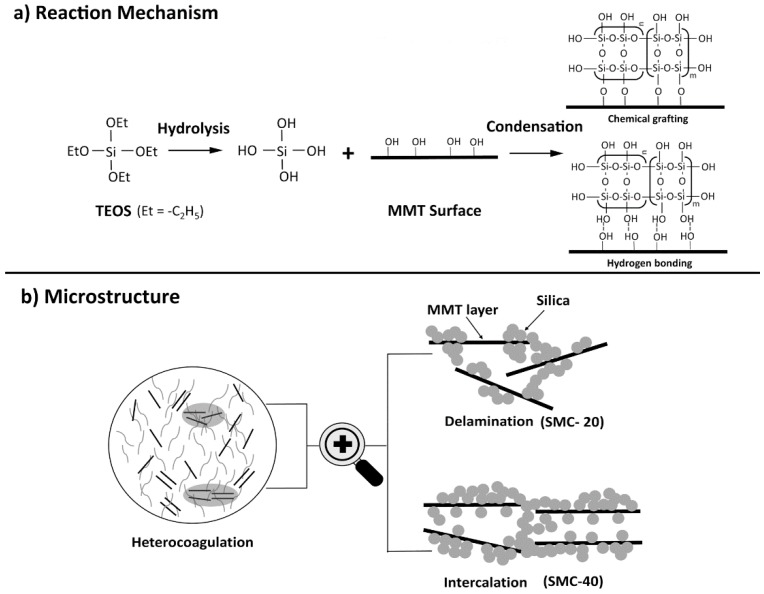
Schematic of reaction mechanism (**a**) and microstructure (**b**) of silica/MMT nanocomposites.

The reaction mechanism and possible heterostructures are illustrated in Figure 7. Intercalation and delamination could be produced depending on the ratio of TEOS to MMT. As shown in Figure 7a, the alkoxide groups (-OC_2_H_5_) could hydrolyze to -OH groups, which could react with the -OH on the clay surface to form chemical bonding or hydrogen bonding like surface modifications of other silicates [41,42]. Therefore, more -OH groups could be grafted onto the MMT surface. It should be pointed out that more TEOS induced intensive self-condensation to form large continuous and complex silica network or silica clusters, especially at high ratio loading levels (e.g., SMC-40). This was because the fast hydrolysis reaction went to produce monosilicic and disilicic acids, which could act as potential nuclei for further colloid particle growth [43]. More nucleation centers led to the formation of a large number of particles. Thereafter, these particles formed networks within minutes at the exterior of the interlayer region of the clay (Figure 7b: intercalation). The degree of this self-condensation could be indicated by the “m” and “n” value (not just a chain propagation but also a three-dimensional network). More complex silica networks (with a bigger “m” and “n” value) preferred to attach to the exterior surface or edge of MMT instead of penetrating into narrow clay galleries. On the other hand, at lower ratios (e.g., SMC-20), the condensation of Si-OH proceeded relatively gradually, which resulted in a simple silica network (oligomers with smaller “m” and “n”). As a result, the silica sol could easily penetrate into the swelling galleries and the MMT clay could be even delaminated (Figure 7b: delamination). 

## 4. Conclusions

Heterostructure silica/MMT nanocomposites were prepared by the organic/inorganic hybrid technique. TEOS, used as a precursor for the silica nanoparticles, was introduced into the interlayer of MMT to prepare the nanocomposites. Different heterostructures of the nanocomposites were obtained by controlling the ratio of TEOS to MMT. A higher ratio formula (40:1) caused the self-condensation of Si-OH, resulting in more nucleation centers before approaching the MMT interlayers, which was negative on the delamination of MMT. Moreover, it was an effective method to introduce MMT clay into a silica matrix to improve the thermal stability of the nanocomposites. The BET specific surface area of the silica/MMT nanocomposites (ca. 474 m^2^·g^−1^) increased with the silica incorporation by showing a decreased pore size (~2.62 nm), indicating the proper agglomeration and porosity of hybrid particles. Compared to pristine MMT, the silica/MMT nanocomposites exhibited advantages such as higher a moisture adsorption, a larger specific surface area and varied porosity. Some of the remained structure of the MMT could be inherited to react with organic compounds via cation exchange capacity in some other fields. Further developments should be explored, focusing on the controlling specific structure and properties based on practical applications.

## Figures and Tables

**Table 1:** Physical parameters of surface structure for MMT and silica/MMT nanocomposites.

Samples	BET Surface Area (m^2^·g^−1^)	Average Pore Width (nm)
MMT	73	4.79
SMC-10	308	3.49
SMC-20	474	2.62
SMC-40	338	3.43
